# Comparison effects of olive leaf extract and oleuropein compounds on male reproductive function in cyclophosphamide exposed mice

**DOI:** 10.1016/j.heliyon.2020.e03785

**Published:** 2020-04-20

**Authors:** Ayoob Rostamzadeh, Hossein Amini-khoei, Mohammad Javad Mardani Korani, Mohammad Rahimi-madiseh

**Affiliations:** a Department of Anatomy, Faculty of Medicine, Iran University of Medical Sciences, Tehran, Iran; b Medical Plants Research Center, Basic Health Sciences Institute, Shahrekord University of Medical Sciences, Shahrekord, Iran

**Keywords:** Spermatogenesis, Olive leaf extract, Cyclophosphamide, Mice, Oleuropein, Cell biology, Plant biology, Pharmaceutical science, Pathophysiology, Laboratory medicine

## Abstract

Spermatogenesis is a complicated process in which sperm is susceptible to various chemotherapy drugs such as cyclophosphamide (CP). As olive leaf extract (OLE) and its active ingredient, oleuropein, have variousantioxidant, anti-apoptotic, and anti-inflammatory properties the aim of the present study was to investigate the effects of OLE and oleuropein on male reproductive function focusing antioxidative effects and histological modifications in the testes of CP-exposed mice.

In order to do this, 80 NMRI male mice were divided into eight groups including control group, group received CP, group received OLE, group received oleuropein, group received OLE following CP exposure, group received oleuropein following CP exposure, group received OLE plus oleuropein and group received OLE plus oleuropein following CP exposure. In all groups CP (single dose of 100 mg/kg (, OLE (100 mg/kg for consequence 28 days) and oleuropein (100 mg/kg for consequence 28 days) were injected intraperitoneally. Moreover, testis histology, sperm parameters and serum levels of LH, FSH, MDA and antioxidant capacity were investigated. Results showed that CP caused oxidative state and abnormal changes in sperms and testes. Besides, treatments with oleuropein and OLE led to mitigate the harmful effects of CP on the male reproductive system. In conclusion, our findings showed that olive's compounds can diminish the hazardous effects of CP on spermatogenesis in mice.

## 1 Introduction

Cyclophosphamide (CP) is an anticancer drug that is converted into an active alkylating metabolite in the body [1]. CP converted to phosphoramide mustard and acrolin. Phosphoramide mustard is responsible for the anticancer properties of CP, but acrolein interferes with the antioxidant defense system and produces large amounts of oxidants [2]. Oxygen-free radicals are responsible for the toxic effects of CP such as cell death, apoptosis, necrosis and formation of various tumors [1, 2].

Olive is an evergreen tree belonging to the oleracea family. Among different parts of the olive tree, fresh leaves have the highest antioxidant activity [3, 4]. Oleuropein, as the most abundant compound in olive leaf extract (OLE), has antioxidant activity [4]. It has been shown that Oleuropein exerted antihypertensive, anti-inflammatory, hypoglycemic, and antiarrhythmic effects [4, 5, 6, 7]. Oleuropein in OLE increases GnRH and testosterone secretion [8, 9]. In addition, the acidic compounds in OLE inhibit the activity of the aromatase enzyme which consequently increases androgens (testosterone and dihydrotestosterone) in the body [10, 11].

The use of chemotherapy drugs has significant negative effects on the reproductive system especially gonads [12]. Usage of medicinal plants to mitigate these harmful effects and treat certain conditions such as hormonal imbalances, oligospermia, low sperm motility, and prostate inflammation has long been considered [13].

Due to the susceptibility of the spermatogenesis process, the administration of CP may decrease sperm count [14]. Besides, Cyclophosphamide led to damaging to spermatogenesis-related stem cells and the destruction of spermatogonial cells [6, 15].

Considering various pharmacological effects of olive and oleuropein and hazardous effects of CP on spermatogenesis, this study aimed to evaluate the protective effects of these compounds on male reproductive function in mice exposed to CP focusing on its antioxidant properties.

## 2 Materials and methods

### 2.1 Plant preparation and extraction

Fresh leaves were gathered from Shaft village, Gilan province, North of Iran on April 2018. Moreover, after identifying the herb by an expert botanist (Shahrokhi, A, M.S., Research Center of Agriculture and Natural Resources, P.O. Box 415, Shahrekord, Iran); it was deposited to the Herbarium Unit of Shahrekord University of Medical Sciences (Herbarium No. 1020). They were dried in the shade away from sunlight at 25 °C in laboratory. To extract the dried and pulverized sample, ethanol 70% was used by maceration method. In the next step, After 72 h, using Whatman filter paper and Buchner funnel, the liquid was filtered and the obtained extract was concentrated in a rotary vacuum evaporator at 38 °C. Furthermore, the concentrated extract was incubated at 37 °C for final drying. The oleuropein compound was also purchased from Sigma (Lot No. 12247, St. Louis, MO, USA).

### 2.2 Animals and grouping

In this experimental study, 80 adult NMRI mice weighing approximately 30 g and aged 8–12 weeks were divided into eight groups:

group 1 (control): received no treatment or injection, group 2: received a single dose of CP, group 3: received OLE, group 4: received oleuropein, group 5: received OLE after CP exposure, group 6: received oleuropein after CP exposure, group 7: received OLE + oleuropein; and group 8: received OLE + oleuropein after CP exposure.

CP was injected intraperitoneally at a single dose of 100 mg/kg. OLE (100 mg/kg) and oleuropein (100 mg/kg) were also administered intraperitoneally in the respective groups for 28 constant days [16]. Doses of agents were chosen based on previously published studies as well as our pilot study [17, 18, 19]. All stages of experimentation were performed in accordance with the regulations of the University and the Guide for the Care and Use of Laboratory Animals of the National Institutes of Health (Ethics code: IR. SKUMS.REC.1397.30).

### 2.3 Measurement of serum parameters

At the end of the study, the animals were anesthetized and blood samples were collected in microtubes and then centrifuged. Standard ELISA kits were used to measure FSH and LH levels [20, 21]. Setiawan's procedure was used to measure serum malondialdehyde (MDA) levels [22]. Serum antioxidant capacity was evaluated by the ferric ion-reducing system (FRAP) [23].

### 2.4 Investigation of sperm parameters

Sperm parameters including sperm count, motility, viability and morphology were investigated according to the procedures used in a previous study. Epididymes were kept in the Ham's F10 medium. Then slicing with a scissor in an incubated at 37 °C for 5 min in order to release of sperms, using eosin-necrosis (E&N) staining, the percentage of live sperms was determined under a microscope. To investigate the sperm morphology, a drop of 20 μl of sperm suspension was spread on a slide and after drying, Aniline-blue staining was performed to determine sperm morphology under a light microscope at a magnification of 400. 10 slides were examined from each animal and parameters including head abnormalities, midpiece defects, and faulty tail were assessed. To assess sperm motility, a small drop of the sample was placed on the slide, and then examined at a magnification of 400 under the microscope. The percentage of motile spermatozoa in several microscope fields was estimated and reported as an average of percentage. In order to evaluate the sperm count, sperm suspension was diluted in 3% normal saline in a proportion of 1–9, and then a drop of the above solution using a micropipette was gently transferred to the neobar slide. After 5 min using a magnification of 40 spermatozoa with head, middle and tail pieces were counted [20, 21, 24].

**Figure 1:**
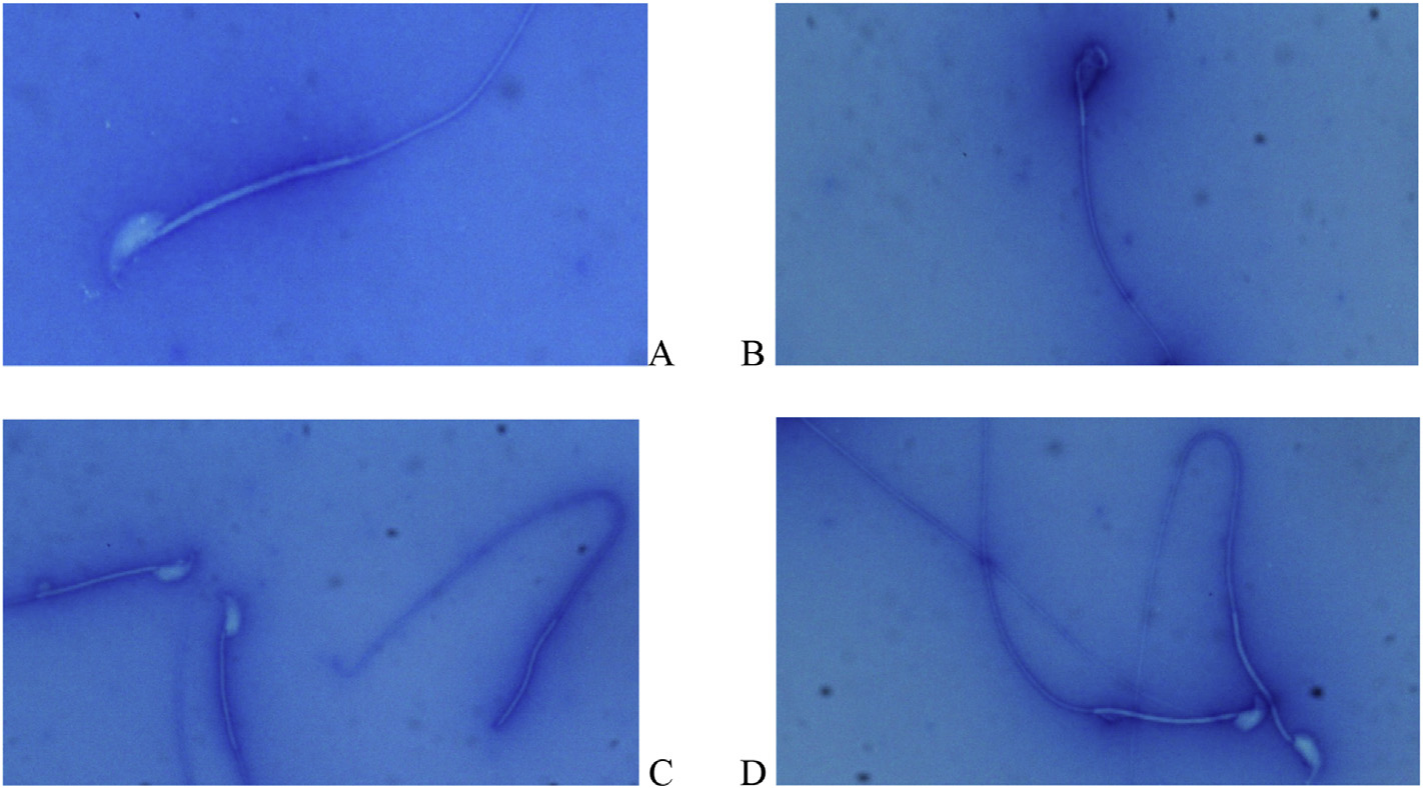
Assessment of sperm morphology using aniline blue staining: A) Healthy sperm with perfectly transparent head and extended and regular tail; B) Sperm with curved midpiece abnormalities that remove the head from the tail axis and have low sperm motility; C) Sperm with amorphous (headless) abnormality that is dead and not able to fertilize the egg; D) Sperm with curved tail, cytoplasmic droplet tail and short tail that disrupt sperm motility and decrease cell viability.

Sperm motility was evaluated according to the following 3 grades: a) grade 1 (immotile): sperms which have no motility; b) grade two (in situ or non-progressive): Sperm whose tails move but have no forward motion; (c) grade Three: Sperm that swim rapidly in a straight forward direction [24]. To evaluate sperm morphology, three abnormal sperms including head abnormalities, midpiece defects, and faulty tail were divided into two groups (Figure 1) [25].

### 2.5 Histological evaluation

In all groups, the right testes were histologically studied by hematoxylin-eosin (H & E) staining. To evaluate the quantitative parameters of seminiferous tubules, linear gradients on the microscope lens were used. To do this, twenty seminiferous tubules in round or nearly round cross-sections were randomly selected from each sample. Twenty cells per each animal were studied. Based on the morphology of each cell, spermatogonia, primary spermatocytes, round spermatids and spermatozoa were counted in the cross-sections of 20 seminiferous tubules per animal. Only round tubules were counted, and tubes that had an inclined section or were elliptical or contained cells that had fallen into the lumen were not counted. Tubules that were elliptical or had inclined sections were not examined for studying seminiferous tubules. At 400× magnification, the diameters of the seminiferous tubules were examined so that the distance between the basement membrane on one side of the tubule and the basement membrane of the other side was measured.

Two diameters perpendicular to each other were measured, and then the mean length of the diameters in each tubule was calculated. Similarly, the mean diameter inside the seminiferous tubule and the thickness of the germinal epithelium were measured in micrometer [26].

### 2.6 Statistical analysis

The data were entered into SPSS version 20 and analyzed using one-way ANOVA and Tukey's post-test. The significance level was considered at level of P < 0.05.

## 3 Results

### 3.1 Results of serum parameters

The results showed that serum FSH level were not significantly different between the CP group and the control group. However, serum FSH levels were significantly higher in healthy mice received the OLE plus oleuropein than in the control group (*P* < 0.001) (Table 1). Serum LH levels significantly decreased in the CP- received group in comparison with the control group (*P* < 0.001). Furthermore, a significant difference in LH level was also observed between CP- received group which treated with OLE + oleuropein (*P* < 0.05). Serum antioxidant capacity in the CP-received group was significantly lower than control group (*P* < 0.001). Serum MDA levels were significantly higher in the CP-treated group than in the control group (*P* < 0.001). Besides, MDA levels were significantly lower in groups received oleuropein and/or OLE in compared with the CP-received group (*P* < 0.001 and P < 0.05, respectively). In addition, simultaneous administration of the OLE and oleuropein decreased MDA levels in comparison with CP-received group (*P* < 0.01) (Table 1).

**Table 1:** Quantitative comparison (mean ± SD) of FSH, LH, antioxidant capacity and MDA levels. Samples were taken from 10 animals in each group. Data were analyzed using one0way ANOVA followed by tukey's post test. ∗∗P < 0.01 AND ∗∗∗P < 0.001compared with control group and #P < 0.05 and ##P < 0.01 compared with CP-received group.

Parameter Groups	Antioxidant capacity	MDA	FSH	LH
1- Control	297.6 ± 1.68	212.1 ± 2.96	4.68 ± 0.31	4.26 ± 0.1
2- CP	183.4 ± 1.61∗∗∗	354.6 ± 3.47∗∗∗	3.11 ± 0.2	2.45 ± 0.16∗∗∗
3- OLE	312.7 ± 2.11	2.9 ± 2.83	6.31 ± 0.52	4.31 ± 0.08
4- oleuropein	298.2 ± 1. 49	210.7 ± 3.09	6.31 ± 0.57	4.28 ± 0.11
5- CP + OLE	197.6 ± 2.36	319.4 ± .07##	3.73 ± 0.25	3.11 ± 0.21
6- CP + oleuropein	191.5 ± 1.11	329.8 ± 2.67#	3.21 ± 0.21	2.81 ± 0.23
7- OLE + oleuropein	321.6 ± 1.54	189.3 ± 4.24	7.56 ± 0.37∗∗∗	4.6 ± 0.24
8- CP + OLE + oleuropein	214.4 ± 2.52	298.1 ± 2.62##	4.48 ± 0.24	3.36 ± 0.16#

### 3.2 Sperm parameters

The results showed that CP exposure reduced sperm count in comparison with the control group (*P* < 0.001). OLE treatment in healthy mice had a significant effect on the increase in sperm count in comparison with the control group (*P* < 0.05). In addition, simultaneous treatment with OLE and oleuropein significantly increased sperm count in comparison with the control group (*P* < 0.01). Besides that, simultaneous treatment of the OLE plus oleuropein in CP-treated mice significantly increased sperm count in comparison with group received CP (*P* < 0.01) as well as in compared to the control group (P < 0.01).

***Motility:*** Grade 1 (immotile): The results showed that CP exposure significantly increased the percentage of immotile sperm in comparison with control group (*P* < 0.001). Grade 2 (in situ): CP- received group showed a significant increase in the count of sperm with in situ motility in comparison with the control group (*P* < 0.01). In group received OLE plus oleuropein situ sperm motility significantly reduced in comparison with CP-received group (*P* < 0.01). Grade 3 (progressive): The results showed that CP exposure, in comparison with the control group, significantly reduced the percentage of progressive motility (*P* < 0.05). The results showed a significant increase in progressive sperm count in group received OLE plus oleuropein in compared with the CP-received group (*P* < 0.05) (Table 2).

**Table 2:** Quantitative comparison (mean ± SD) of sperm count and motility. Samples were taken from 10 animals in each group. Data were analyzed using one0way ANOVA followed by tukey's post test.∗p < 0.05, ∗∗P < 0.01 and ∗∗∗P < 0.001compared with control group and #P < 0.05 and ##P < 0.01 compared with CP-received group.

Parameter Groups	Sperm Count	Sperm motility		
		Immotile	In situ	Progressive
1- Control	75.5 ± 2.21	4.8 ± 0.8	4.8 ± 0.8	42.2 ± 1.74
2- CP	45.5 ± 1.25∗∗∗	9.4 ± 0.56∗∗∗	10.9 ± 0.52∗∗	32.5 ± 1.35∗
3- OLE	84.2 ± 1.6∗	4 ± 0.25	4.4 ± 0.47	46 ± 1.43
4- oleuropein	79.5 ± 1.9	4.5 ± 0.52	4.8 ± 0.46	44.7 ± 1.17
5- CP + OLE	56 ± 1.94	7.7 ± 0.59	8.7 ± 0.44	36.7 ± 1.01
6- CP + oleuropein	49.7 ± 1.51	8.8 ± 0.38	9.6 ± 0.26	34.7 ± 1.23
7- OLE + oleuropein	86.5 ± 1.8∗∗	3.6 ± 0.4	3.6 ± 0.6	50.5 ± 2.57
8- CP + OLE + oleuropein	59.2 ± 1.34##,∗∗	7.2 ± 0.71	7.6 ± 0.4##	39.8 ± 1.2#

***Viability:*** We showed that CP exposure group significantly reduced the percentage of viable sperm in comparison with the control group) (*P* < 0.0001) (Table 3).

**Table 3:** Quantitative comparison (mean ± SD) of viability and morphology of sperm. Samples were taken from 10 animals in each group. Data was analyzed using one0way ANOVA followed by tukey's post test. ∗∗∗P < 0.001compared with control group and ##P < 0.01 and ###P < 0.001 compared with CP-received group.

Parameter Groups	Viable sperm	Sperm morphology		
		Head abnormalities	Midpiece defects	Faulty tail
1-Control	61 ± 1.09	1.1 ± 0.23	2.5 ± 0.45	2 ± 0.36
2- CP	36.2 ± 1.44∗∗∗	5.5 ± 0.42∗∗∗	6.2 ± 0.64∗∗∗	7.4 ± 0.63∗∗∗
3- OLE	63.7 ± 3.14	0.8 ± 0.2##	1.7 ± 0.36	1.6 ± 0.3
4- oleuropein	62.1 ± 1.2	0.9 ± 0.17	2.1 ± 0.27	1.8 ± 0.35
5- CP + OLE	40.7 ± 1.53	3.7 ± 0.49	4.9 ± 0.45	5.2 ± 0.51
6- CP + oleuropein	38.4 ± 0.73	4.7 ± 0.33	5.7 ± 0.47	6.7 ± 0.9
7- OLE + oleuropein	64.5 ± 1.24	0.4 ± 0.16	1.2 ± 0.29	1.1 ± 0.27
8- CP + OLE + oleuropein	48.5 ± 1.33##, ∗∗	3 ± 0.25###	3.8 ± 0.48##	4.4 ± 0.54

Results showed that simultaneous administration of the OLE with oleuropein significantly increased the count of viable sperm in comparison with the CP- received group (*P* < 0.01) as well as in compared to the control group (P < 0.01).

***Morphology:*** Head Abnormalities: The results showed that CP exposure significantly increased sperm with abnormal head morphology in comparison with the control group (P < 0.001). Administration of the OLE significantly decreased sperm with abnormal head in comparison with the CP-received group (*P* < 0.01). Simultaneous administration of the OLE with oleuropein significantly decreased sperms with abnormal head in comparison with Cp-received counterpart (*P* < 0.001). Midpiece defects: The results showed a significant increase in midpiece defects in CP-received group in comparison with the control group (*P* < 0.0001). Furthermore, co-administration of OLE with oleuropein significantly decreased sperm with midpiece abnormalities in comparison with CP-received counterpart (*P* < 0.01) (Table 3). Faulty tail sperm: The results showed that the percentage of sperm with faulty tail significantly increased following CP administration in comparison with the control group (*P* < 0.001).

### 3.3 Qualitative histological results

**Figure 2:**
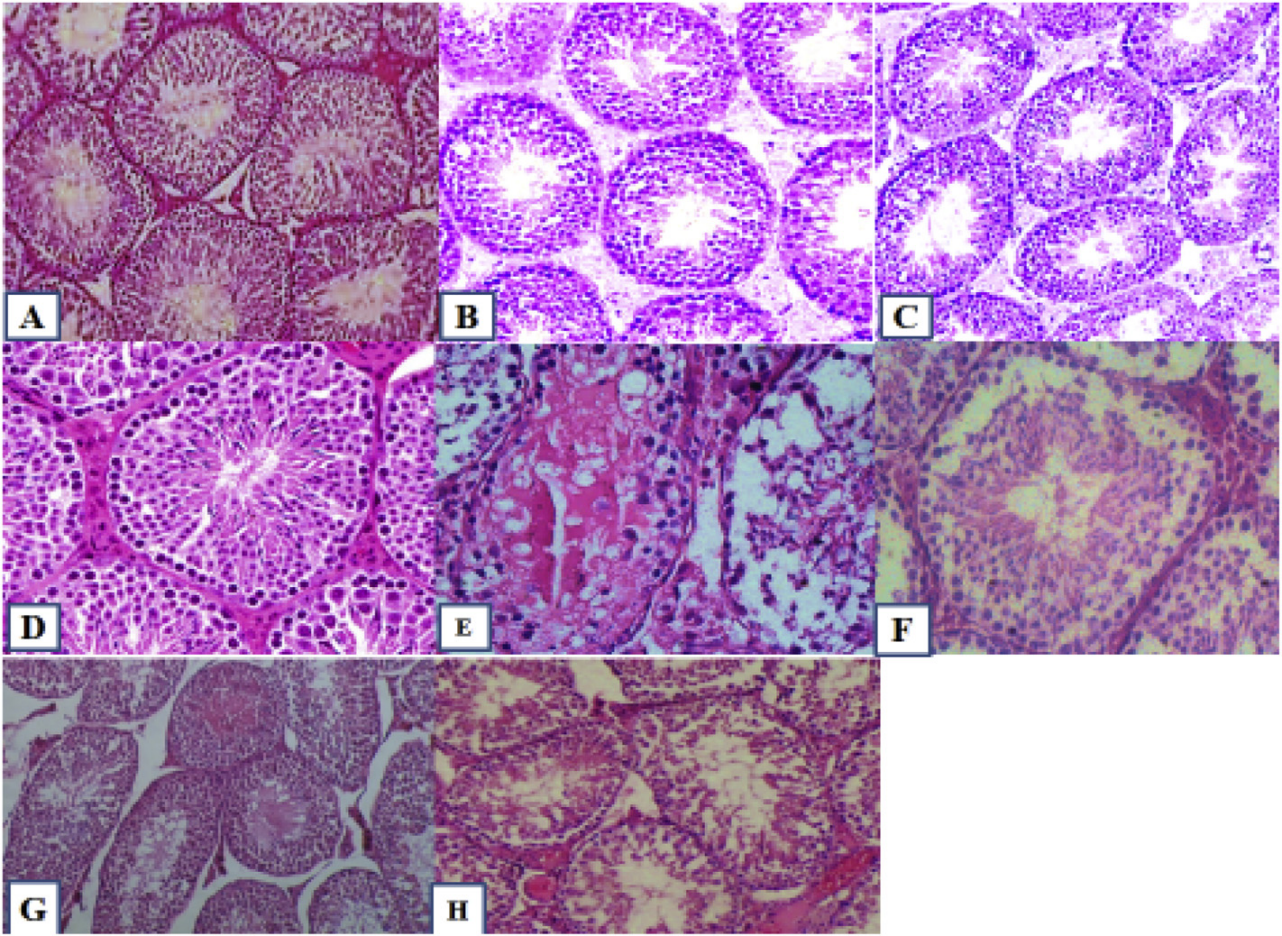
The testicular histopathological investigations in experimental groups showing different degrees of degenerative changes in testicular tissue: A) Group 1 (control); B) Group 3 (treated with OLE alone); C) Group 4 (treated with oleuropein alone); D) Group 7 (simultaneously treated with OLE and oleuropein without cyclophosphamide administration); E) group 2 (treated with cyclophosphamide alone); F) Group 5 (treated with OLE and cyclophosphamide); G) group 6 (treated with oleuropein and cyclophosphamide); H) Group 8 (simultaneously treated with OLE and oleuropein with cyclophosphamide administration). Please note that all figures are presents as supplementary file.

The results of histopathologic examination of the testis in different groups show different and varying degrees of degenerative changes in the testicular tissue (Figure 2). Following CP administration, the diameter of the seminiferous tubules decreased, the seminiferous tubules were irregular and the basement membrane was crimped, the epithelium also had severe atrophy and vacuolization as well as spermatogenesis was not seen at all stages. In CP- received group, primary spermatocytes at pachytene stage and arrest were immotile. There was substantial edema and inflammation along with a decrease in the number of seminiferous tubules (Figure 2 E). Our findings showed that administration of OLE, oleuropein and their combination therapy partially mitigated the negative effects of CP on testicular tissue.

## 4 Discussion

Spermatogenesis is a long and complex process, during which the sperm is sensitive to various internal and external stressors [27]. The most common cause of infertility in men is their inability to produce adequate number of healthy and active sperms [28, 29]. The administration of chemotherapy drugs lead to harmful effects on the gonads and its damage to spermatogonia-producing germ cells which consequently caused infertility [12]. Cyclophosphamide (CP) is an anticancer drug that is converted into an active alkylating metabolite in the body leading to hazardous effects on male gonads [30]. Meistrich *et al.* (2013) showed that CP at therapeutic doses decreased sperm count in patients [31]. Moreover, a study by Elangovan *et al.* (2006) on the reproductive system of mice exposed to CP showed that this drug reduced the blood LH and FSH levels [32]. The present study showed that CP decreased the sperm parameters as well as decreased levels of sex hormones.

Flavonoids are natural polyphenolic compounds in OLE that exerted anti-inflammatory effects by reducing the production of nitric oxide and prostaglandins [33]. Oleuropein has high antioxidant power which can be found in olive oil and is able to exert anti-inflammatory and antioxidant effects [34]. Previous studies have been determined that OLE significantly increased spermatogenesis and sperm quality [35]. A study by Sarbishegi *et al.* (2017) showed that oleuropein increasedsperm parameters. Increased total antioxidant capacity (TAC) as well as decreased malondialdehyde (MDA) level in the testes in animals received rotenone (ROT) were reported [36]. The findings of our study showed that injection of OLE in mice exposed to CP relatively had greater antioxidant, protective, and even regenerative effects than oleuropein. In addition, simultaneous injection of OLE in combined with oleuropein increases LH levels and decreases MDA levels in CP-treated rats as well as improves the histological characteristics of seminiferous tubules. Based on our findings, future studies warranted might also find more mechanisms of action of these compounds.

It has been determined that oxidative states like increase in the MDA level and/or decrease in antioxidant capacity negatively affected spermatogenesis [37, 38]. Furthermore, high levels of oxidants result in the destruction of bases, cross-linking of proteins, and DNA breakdown and consequently activates apoptotic cascade reactions [39, 40, 41]. Our findings also showed that administration of CP increased the MDA level as well as decreased the antioxidant capacity. We found that co-administration of OLE + oleuropein significantly mitigated these negative effects. It has been determined that aromadendrine, a component of the flavonoid pattern of Olea europaea L. possessed anti-inflammatory activity [42]. Further studies must be conducted in the future to find the exact mechanisms of action of the oleuropein and OLE and its active components like aromadendrine in this field.

## 5 Conclusion

The results of the present study showed that cyclophosphamide caused significant alterations in the testes tissue, negatively affected the sperms' parameters (number, mobility and morphology) as well as changed the level of sex hormones. We found that administration of OLE and oleuropein exerted protective effects against CP- induced negative alterations in the male reproductive system in mice.

## Declarations

### Author contribution statement

M. Rahimi-Madiseh: Conceived and designed the experiments; Analyzed and interpreted the data; Contributed reagents, materials, analysis tools or data; Wrote the paper.

A. Rostamzadeh: Conceived and designed the experiments; Performed the experiments; Wrote the paper.

H. Amini-khoei: Performed the experiments; Analyzed and interpreted the data; Wrote the paper.

M. J. M. Korani: Performed the experiments.

### Funding statement

This research did not receive any specific grant from funding agencies in the public, commercial, or not-for-profit sectors.

### Competing interest statement

The authors declare no conflict of interest.

### Additional information

Data is deposited in the Medical Plants Research Center, Basic Health Sciences Institute, Shahrekord University of Medical Sciences, Shahrekord, Iran.

Supplementary content related to this article has been published online at https://doi.org/10.1016/j.heliyon.2020.e03785.

## Appendix A Supplementary data

Supplementaryu file Supplementaryu file
