# Supplementary figures and images for: Comparison effects of olive leaf extract and oleuropein compounds on male reproductive function in cyclophosphamide exposed mice
Source: Heliyon. 2020 Apr 20;6(4):e03785. doi: 10.1016/j.heliyon.2020.e03785 (PMC7176941; doi:10.1016/j.heliyon.2020.e03785)

Fig 2A


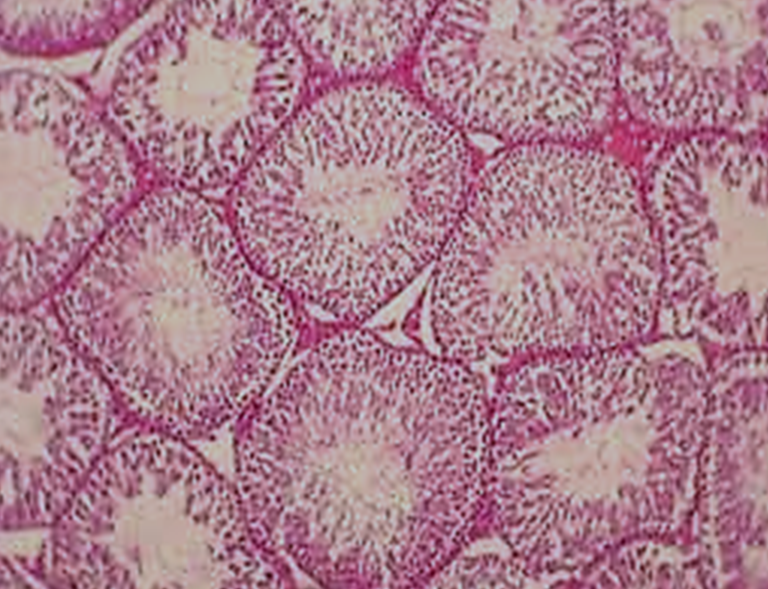


Fig 2B


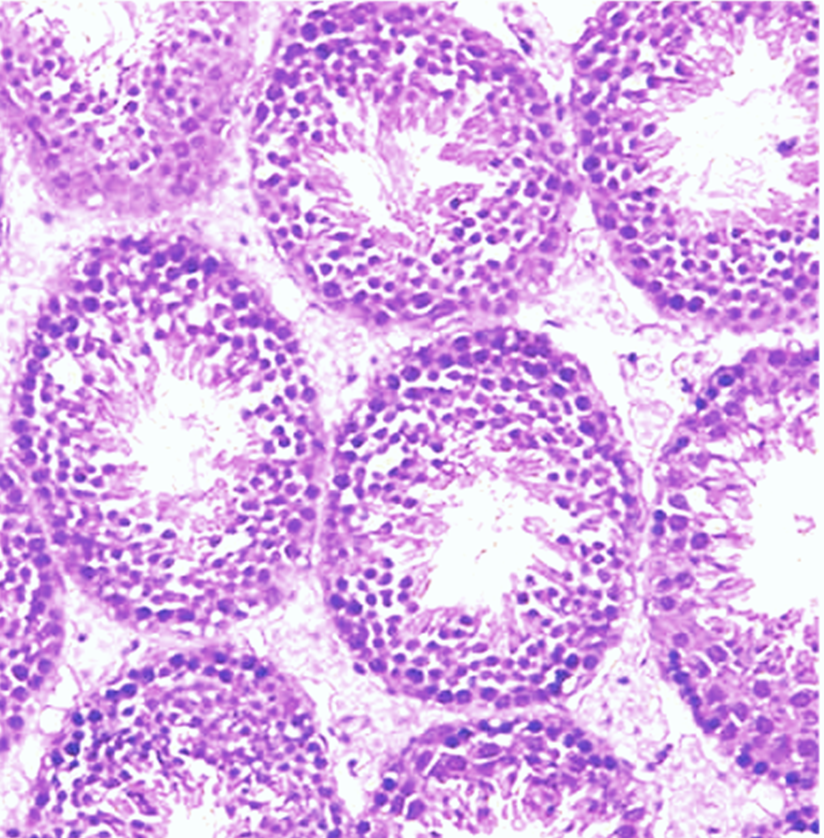


Fig 2C


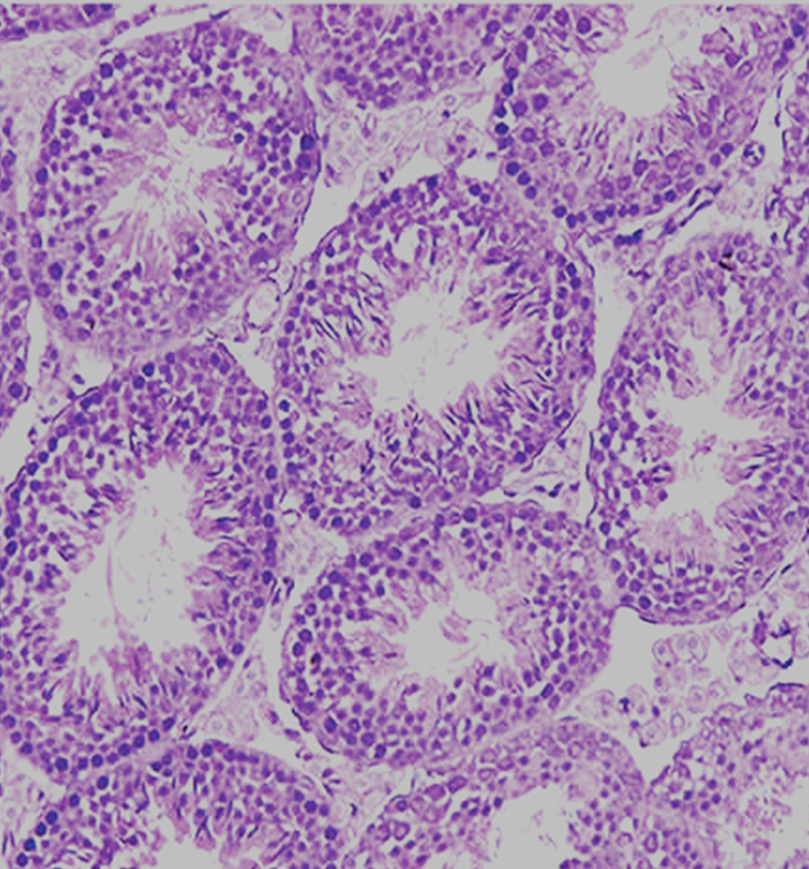


Fig 2D


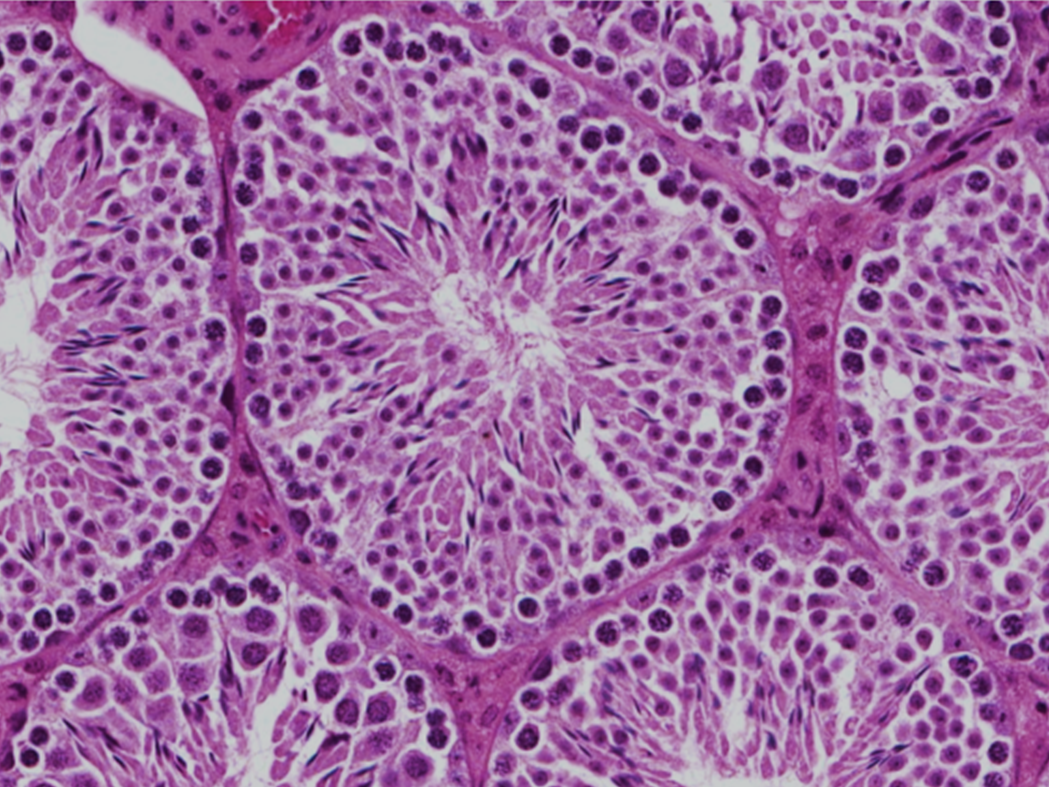


Fig 2E


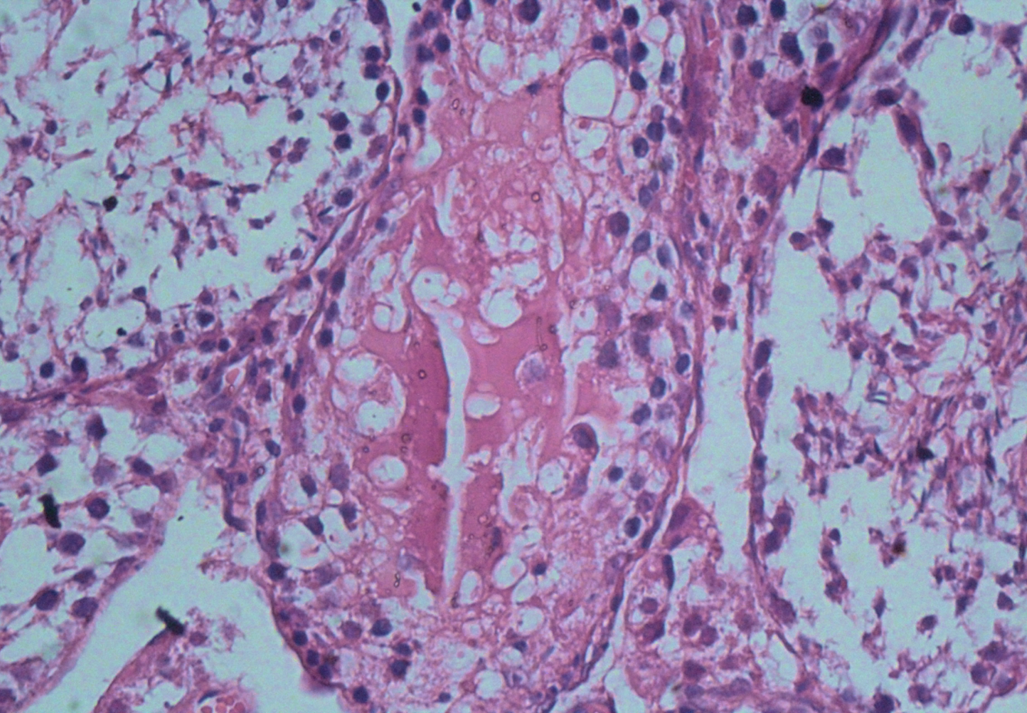


Fig 2F


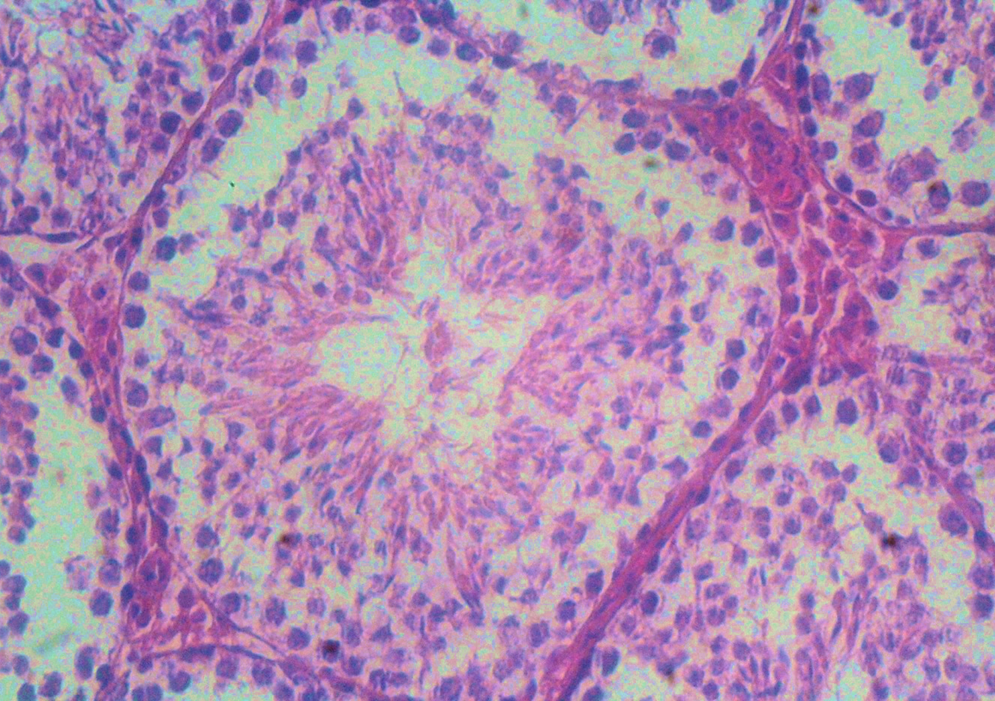


Fig 2G


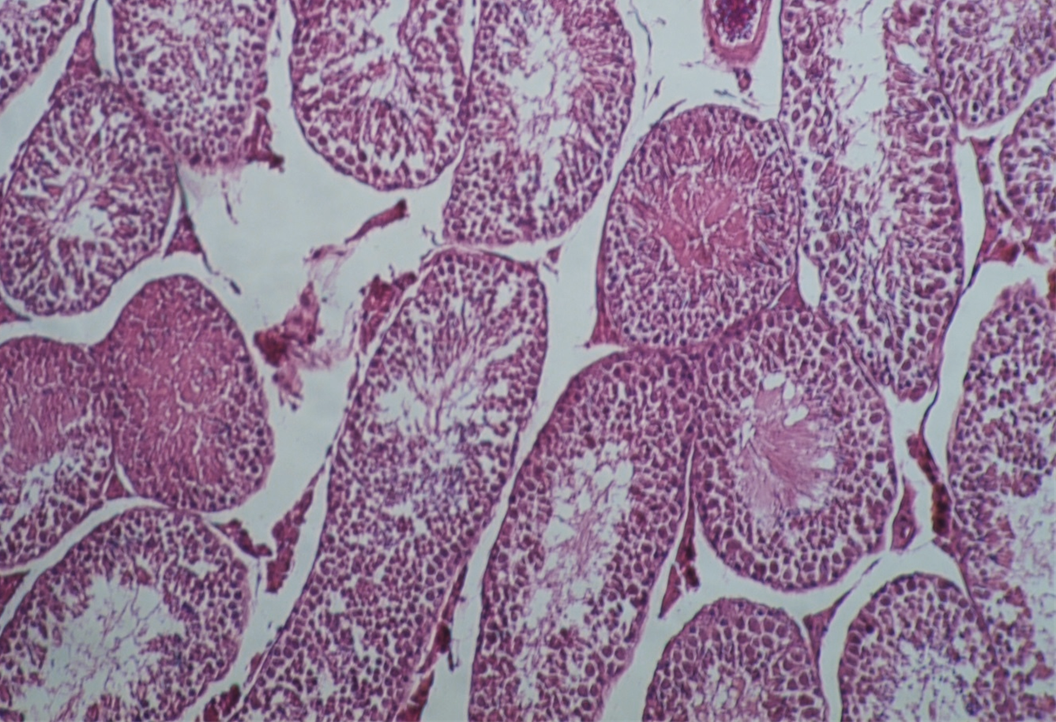


Fig 2H


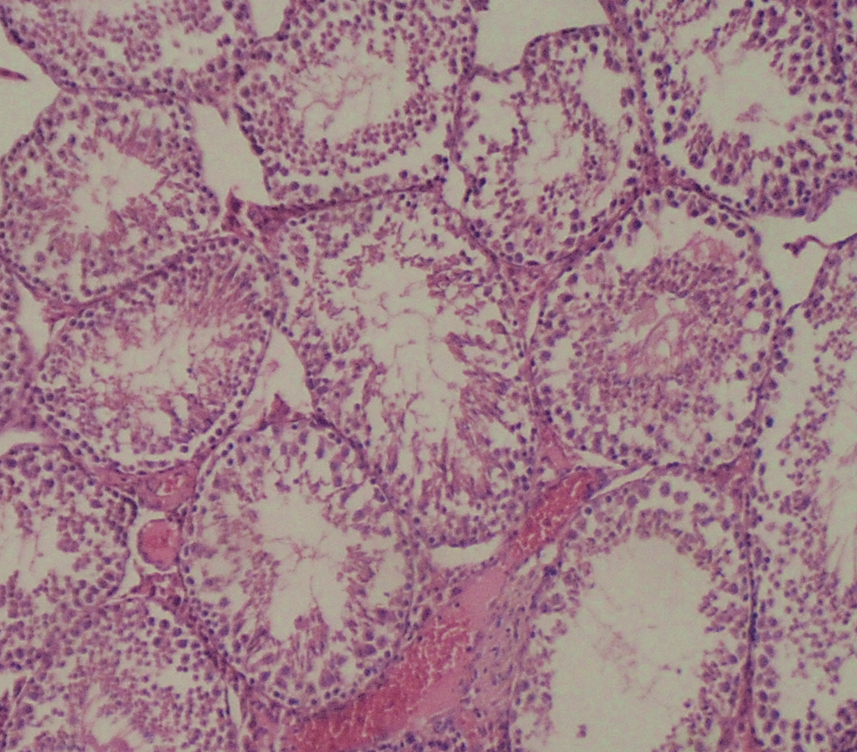

Supplement: Supplementaryu file [file mmc1.docx]
